# Sex Determines Anterior Cingulate Cortex Cortical Thickness in the Course of Depression

**DOI:** 10.1016/j.bpsgos.2023.08.003

**Published:** 2023-08-13

**Authors:** Guo-Rong Wu, Chris Baeken

**Affiliations:** a Key Laboratory of Cognition and Personality, Faculty of Psychology, Southwest University, Chongqing, China; b Ghent Experimental Psychiatry Lab, Faculty of Medicine and Health Sciences, Department of Head and Skin, Ghent University, Ghent, Belgium; c Ghent Experimental Psychiatry Lab, Faculty of Medicine and Health Sciences, Department of Head and Skin, Ghent University, Ghent, Belgium; d Department of Psychiatry, University Hospital (Universitair Ziekenhuis Brussel), Vrije Universiteit Brussel, Brussels, Belgium; e Department of Electrical Engineering, Eindhoven University of Technology, Eindhoven, the Netherlands

**Keywords:** ACC, Cortical thickness, First episode depression, Major depressive disorder, Recurrent depression, Sex

## Abstract

**Background:**

Major depressive disorder (MDD) is a severe psychiatric disorder affecting women more than men. Changes in anterior cingulate cortex cortical thickness (ACC CT) may be crucial to understanding sex influences in MDD onset and recurrency.

**Methods:**

Taken from the large open-source REST-meta-MDD database, we contrasted 499 patients with MDD (381 first-episode MDD, 118 recurrent MDD) and 524 healthy control participants using linear mixed-effects models and normative modeling and investigated whether sex differences affected ACC CT and its subregions differently during the course of depressive illness.

**Results:**

Overall, females showed thinner ACC CT compared with males. Female patients with a first depressive episode showed significantly thinner ACC CT compared with male patients with first-episode MDD (Cohen’s *d* = −0.65), including in the perigenual ACC and the subgenual ACC, but not in the dorsal ACC. Moreover, male patients with first-episode depression showed thicker ACC CT (including subgenual ACC and pregenual ACC) compared to the male patients with recurrent MDD (Cohen’s *d* = 1.24), but they also showed significantly thicker cortices in the same subregions in comparison to never-depressed males (Cohen’s *d* = 0.85). No lateralization differences were observed in ACC CT or its subdivisions.

**Conclusions:**

Sex determined ACC CT changes over the course of depressive illness. Because the ACC subdivisions in question are associated with dysregulation of emotions, our observations substantiate the need for early and prolonged sex-specific clinical interventions.

Major depressive disorder (MDD) is one of the most common mental health problems worldwide, with a high relapse rate and high risk of chronicity, and regardless of country and culture, it is more prevalent in women than in men (1). Furthermore, sex differences have been documented in the presentation of clinical symptoms (2). For example, cross-sectional baseline data from the large NESDA (Netherlands Study of Depression and Anxiety) study showed sex differences in the clinical presentation of MDD with the most notable outcomes in women being a younger starting age, comorbid anxiety, lower alcohol use, and a higher prevalence of atypical depression (3). In contrast, depressed men may be more likely to report externalizing symptoms, such as heightened risk taking, poor impulse control, and substance misuse (4). In addition, it has been suggested that structural, immunological, genetic, and environmental factors as well as stress hormones are also responsible for different clinical symptom presentations between men and women in MDD (5,6).

Although it can be assumed that sex-specific neurobiological and emotional regulation processes are at play, it remains unclear why some men and women are more susceptible to a (first) depressive episode and why some remain vulnerable to depressive relapses even after they have fully recovered (7, 8, 9). No clear structural (or functional) brain biomarkers have been found to date that predict the occurrence of a depressive episode, treatment outcome, or relapse (10,11). Notwithstanding the fact that it is generally recognized that patients with depression may show a decrease in brain volume as early as the onset of the first depressive episode (12,13), increases in brain volume have also been observed, even during the earlier stages of the disorder (14, 15, 16). A recent review showed that sex differences in MDD may also influence structural and functional brain imaging results; however, due to low sample sizes, no firm conclusions were drawn (17).

Cortical thickness (CT), which is the distance between corresponding points on the pial and white-matter boundaries of the neocortex, may provide a better measure of cortical mantle integrity in disease states (18). While Sowell *et al.* found that healthy females displayed thicker CT in the parieto-temporal parts of the brain than males (19), Luders *et al.* showed significantly greater CT in women than in men even after correcting for individual differences in brain size (14). In addition, the few existing studies regarding first-episode versus recurrent MDD are not that straightforward. For example, young women who are at increased risk of MDD (but without current depression) showed thicker (subcallosal) anterior cingulate cortex (ACC) compared with women with depression (20), but it has also been suggested that regardless of sex, larger ACC volumes may be protective against the development of MDD (21). Because remitted patients with depression showed an increase in the ACC and the orbitofrontal cortex, both key regions in emotion regulation (22), it was suggested that an increase in ACC thickness might be associated with a beneficial course of illness or successful treatment and prevention of relapse (21). Furthermore, while structural changes may be more discrete for first-episode depression, for recurrent MDD, more widespread decreases in CT can be expected (23,24); however, it is not clear currently whether sex differences in ACC CT affect the course of depression.

As mentioned before, the ACC is considered an important area in the interplay between affective and cognitive regulatory processes. The more dorsally located parts of the ACC, the dorsal ACC (dACC), is thought to be primarily involved in cognitive regulatory processes, while the pregenual ACC (pgACC) together with the subgenual ACC (sgACC) are more involved in affect regulation (25). For the entire anatomical region, mostly ACC CT decreases have been documented, especially in relation to stress-related disorders such as depression and anxiety (26, 27, 28). Furthermore, it has been shown that adults with MDD exhibited thinner cortical gray matter in the lower regions of the medial prefrontal cortex, including the pgACC and the sgACC (15,24), suggesting that reductions in ventromedial cortical gray matter volumes may be part of the pathophysiology of MDD (29). This is consistent with the sgACC being suggested as a potential biomarker for the (treatment-resistant) depressive state and as a potential clinical predictor of effective antidepressant treatment (30). The dorsal parts of the ACC have also been linked with CT reductions in first-episode depression (31); however, it is not clear whether this is a state or trait effect (32). In addition, given the interplay between affect regulation and cognition within the ACC, it remains unclear which subcomponent(s) of the ACC CT are most affected and at what stage of the course of this psychiatric disorder this may become structurally apparent. Finally, given the possibility that these affective-cognitive functions follow a specific lateral pattern (33,34), it is questionable whether (sub)ACC CT lateralization differences play a role between sexes and during the course of the depressive illness.

To examine these unanswered questions, we first hypothesized that the ACC CT would be significantly thinner in patients experiencing relapse than in patients with first-episode MDD. Given the key roles that they play in stress regulation, we also hypothesized that these ACC CT reductions would primarily be found in the pgACC and sgACC areas. Secondly, given the distinct clinical symptom presentations between the 2 sexes and the potential differences between them in affect regulation, we expected sex-related ACC CT differences primarily among patients experiencing a first depressive episode, especially within the pgACC and sgACC areas. Finally, we explored whether lateralization, medication use, or depression severity influenced (subregion)ACC CT changes in relation to the course of a patient’s depression.

## Methods and Materials

### Participants

**Figure 1:**
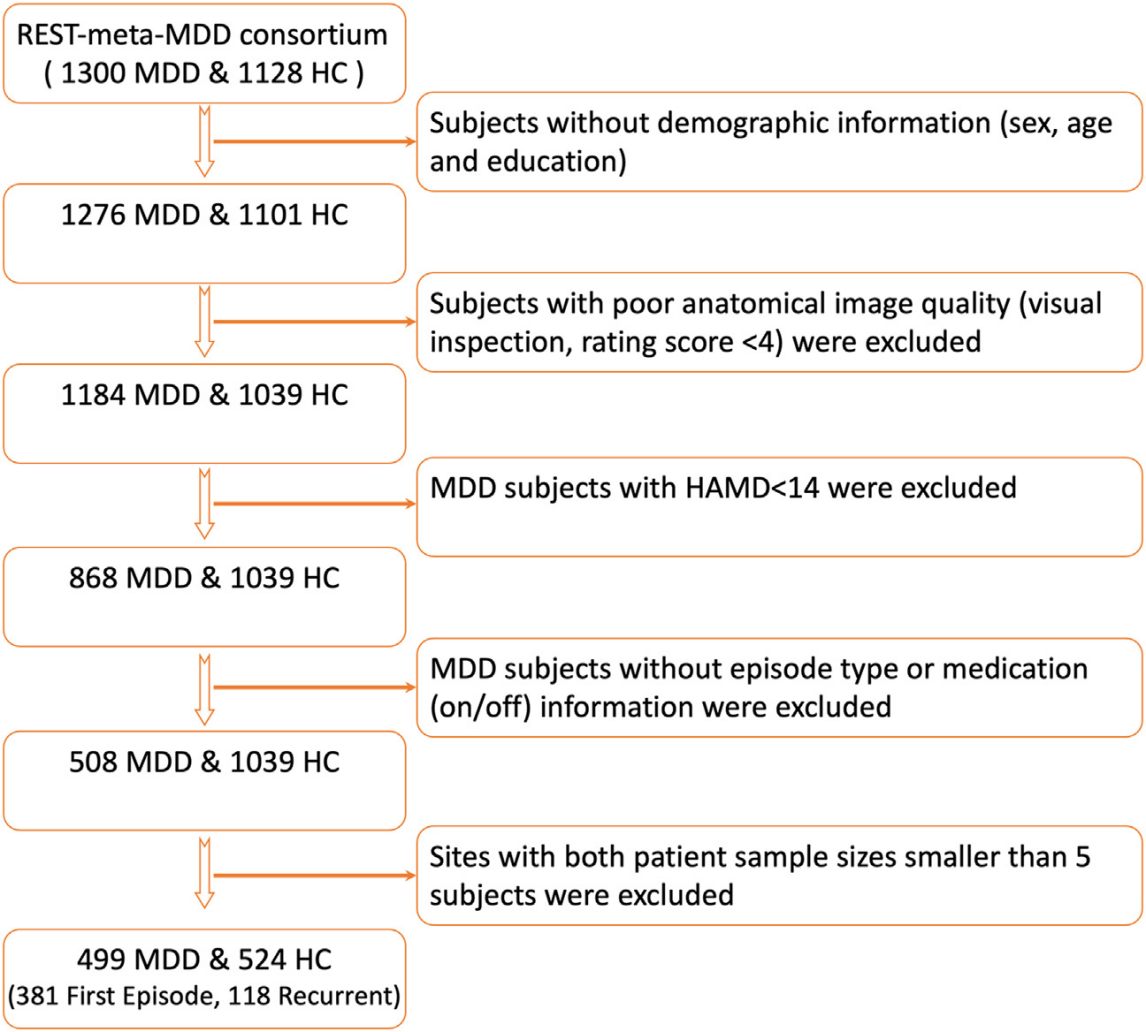
Flow chart with inclusion and exclusion criteria. HAMD, Hamilton Depression Rating Scale; HC, healthy control participant; MDD, major depressive disorder.

Structural brain imaging data were used from the REST-Meta-MDD Consortium (35), which consists of 25 research groups in 17 hospitals in China. Together, the consortium included 2428 collected datasets (1300 currently depressed individuals and 1128 healthy control participants [HCs]; age range, 12–82 years) [for more details on inclusion and exclusion criteria, see (35)]. All patients met the DSM-IV criteria for MDD (36) and were included only if they had a total score of 14 or more on the 17-item Hamilton Depression Rating Scale (HAMD) (37), indicating moderate to severe depression. For the current ACC CT study, based on several quality measures, participants were also excluded for the following reasons: 1) poor image quality (via visual inspection, rating scale score from 1 [poor quality] to 5 [excellent quality], with rating scale score <4); 2) coming from a hospital site with both patient (first-episode MDD, relapsing MDD) sample sizes <5; and 3) no information on medication status. See the flow diagram in Figure 1.

Consequently, 499 patients with MDD (381 first-episode MDD, 118 recurrent MDD) and 524 HCs with an age range of 12 to 77 years were selected from 9 study sites. All study sites obtained approval from their local institutional review boards and ethics committees, and all participants provided written consent at their local institutions.

### Brain Imaging

**Figure 2:**
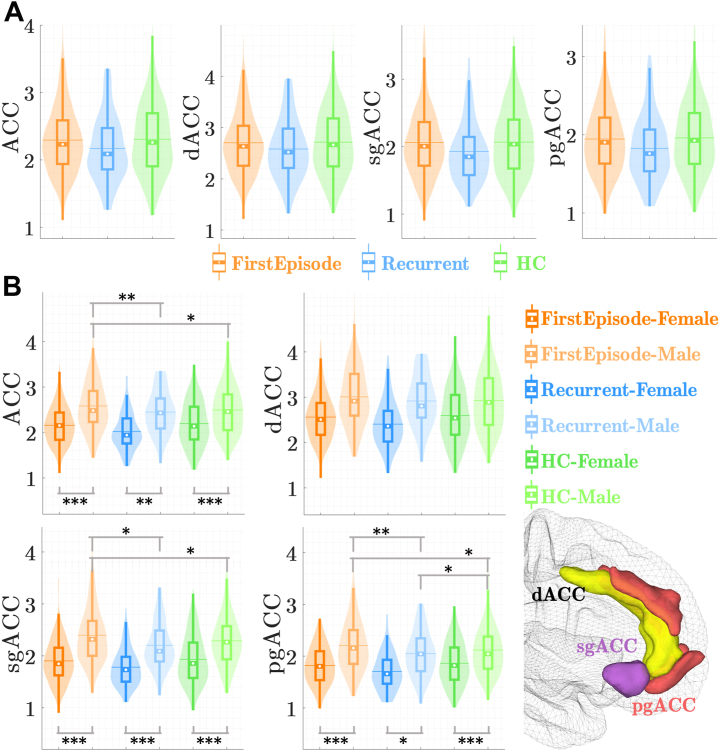
Distribution of anterior cingulate cortex (ACC) cortical thickness across groups and sex. False discovery rate correction was applied for the simple effects tests of group × sex interaction. (∗*p* < .05, ∗∗*p* < .01, ∗∗∗*p* < .001). dACC, dorsal ACC; HC, healthy control participant; pgACC, pregenual ACC; sgACC, subgenual ACC.

Structural T1-weighted magnetic resonance imaging brain scans were acquired at each site [see (35) for magnetic resonance imaging acquisition parameters]. We used the extended Human Connectome Project multimodal parcellation atlas of the human cortex and subcortical areas defining the ACC as Brodmann area (BA) 24 (dACC), BA 25 (sgACC), and BA 32 (pgACC) (see Figure 2).

### Data Analysis

#### Behavioral Data

All behavioral analyses were carried out in the R language. The significance level was set at *p* < .05, two-tailed for all analyses.

#### Imaging Data

The anatomical images were first bias-field corrected and segmented into gray matter, white matter, and cerebrospinal fluid using the SPM12 (http://www.fil.ion.ucl.ac.uk/spm) toolbox. Then, CT estimation was further performed by the DiReCT (diffeomorphic registration-based cortical thickness) algorithm available in ANTs (Advanced Normalization Tools) (38). As suggested by Schwarz *et al.* (39), SPM12 segmentation input to ANTs thickness (SPM + DiReCT) method was the most reliable of all the thickness-based measures. Finally, the resulting CT maps were modulated and resampled into 2 × 2 × 2 mm using the DARTEL (Diffeomorphic Anatomical Registration using Exponentiated Lie algebra) algorithm (40). Quality controls of all CT images were conducted before further statistical analysis.

#### Statistical Analysis

To control for site-related confounding, we employed the linear mixed-effect (LME) model implemented with the 3dLMEr program within AFNI (Analysis of Functional NeuroImages) (41):

(1) ACCCT∼1+Group+Sex+Group:Sex+Age+Education+(1+Group|Site)

where “:” is the Wilkinson-Rogers notation, and Group:Sex indicates the interaction between group and sex. In this model, the intercept and the group variable (first-episode MDD vs. recurrent MDD vs. HCs) contained random effects that were specific to site and fixed effects that were independent of site. Other variables were considered covariates of no interest. Given that sex- and age-related differences in the morphology of the CT have been reported [e.g., (14,42,43)] and that intelligence may influence CT [e.g., (44)], we added sex, age, and education as covariates in the analysis. Post hoc/simple effects tests with Benjamini–Hochberg false discovery rate (FDR) correction (45) were further performed within the 3dLMEr routine if the main effect of group or group by sex interaction was significant.

First, to examine our main research question of whether ACC CT is affected differently in first-episode and recurrent MDD patients, we used the entire ACC in this model. Secondly, to examine whether ACC subparts could be affected differently, we repeated the CT analysis with the 3 ACC subdivisions (dACC, pgACC, and sgACC) separately. Lastly, to explore (sub)ACC CT lateralization group influences, we repeated all CT analyses separately for the left and right ACC and for the 3 subdivisions. Type I error due to multiple comparisons across ACC subdivisions (lateralization) was controlled for using Benjamini–Hochberg FDR correction.

To evaluate the effects of depression severity, we used the LME model to describe the relationship between HAMD scores and ACC CT in first-episode and recurrent MDD groups separately, with age, sex, education, and medication (yes/no) as covariates:

(2) ACCCT∼1+HAMD+Age+Sex+Education+Medication+(1|site).

Finally, to assess the within-group heterogeneity, we also applied neuroanatomical normative modeling to capture individual-level variability in ACC CT, performed within the PCNToolkit (version 0.18) package at https://pcntoolkit.readthedocs.io (46). Hierarchical Bayesian regression analysis was employed to accommodate intersite variation and generate normative models using the covariates of sex, age, and education. The deviations (*z* statistics) for each participant that quantify the degree of an individual’s CT differences from the norm were computed and submitted for the above statistical analysis. For these results, see Figures S3–S5.

## Results

### Demographics

The 3 groups (first-episode MDD, recurrent MDD, HCs) did not differ in age or sex. However, education was significantly different between groups (*F*_2,4.87_ = 13.106, *p* = .011, ${\eta_{p}^{}}^{2}$ = 0.84, 95% CI = [0.36–1]), with higher education levels in the control group. Patients with first-episode and recurrent depression did not differ significantly in depression severity scores (HAMD) (*F*_1,22.05_ = 0.008, *p* = .929, ${\eta_{p}^{}}^{2}$ = 0.0004, 95% CI = [0–1]), but more of the patients with first-episode MDD were off medication than the patients with recurrent MDD (*p* < .001, Cohen’s *w* = 0.29, 95% CI = [0.22–1]). For an overview, see Table 1. Notably, no significant group × sex interactions with demographic variables were observed (all *ps* > .05).

**Table 1:** Demographic Variables

	Sex	*n*	Age, Years, Mean (SD)	Education, Years, Mean (SD)	Medication, Yes/No	HAMD, Mean (SD)
FE MDD	Total	381	34.72 (12.26)	11.39 (3.72)	131/250	22.66 (4.78)
	Female	259	35.31 (12.10)	11.31 (3.74)	94/165	22.98 (4.64)
	Male	122	33.45 (12.56)	11.57 (3.68)	37/85	21.96 (5.03)
Recurrent MDD	Total	118	39.98 (13.26)	10.43 (3.87)	81/37	22.86 (5.30)
	Female	77	42.04 (12.69)	10.36 (3.97)	50/27	23.22 (5.19)
	Male	41	36.12 (13.59)	10.56 (3.73)	31/10	22.20 (5.49)
HC	Total	524	36.77 (14.18)	12.89 (4.18)	–	–
	Female	329	37.79 (14.45)	12.51 (4.26)	–	–
	Male	195	35.05 (13.57)	13.54 (3.96)	–	–

Group, *p*/${\eta_{p}^{}}^{2}$	–	–	.236/.15	.011^a^/.84	<.001^b^	.929/.0004
Sex, *p*/${\eta_{p}^{}}^{2}$	–	–	.012^c^/.0067	.211/.0019	.885^b^	.093/.0064
Group × Sex, *p*/${\eta_{p}^{}}^{2}$	–	.27^b^/NA	.720/.00067	.162/.0038	–	.847/.00008

FE MDD, first-episode major depressive disorder; HAMD, Hamilton Depression Rating Scale; HC, healthy control participant.

a Post hoc Tukey’s HSD tests indicated that educational attainment was higher in the HC group relative to the recurrent MDD group (*t*_5.26_ = 3.42, *p*_adjusted_ = .039).

b χ^2^ test of independence; *df*(group) = 2, *df*(sex) = 1.

c Females have a higher mean age than males.

### ACC Cortical Thickness

Overall, CT in the ACC and within its subdivisions did not differ significantly between groups. See Table 2 and Figure 2A. Furthermore, no significant lateralized (sub)ACC CT was observed (Figure S1). Importantly, sex differences were observed over all groups, meaning that women displayed significantly thinner ACC CT compared with men regardless of whether they were experiencing first-episode or recurrent MDD or were nondepressed (HCs) (see Figure 2B). The LME model showed no relationship between HAMD scores and CT in the ACC (or its subdivisions) in the first-episode and recurrent MDD groups separately (all *ps* > .05). Education varied across groups; nonetheless, it did not seem to affect CT (*F*_1,1008.25_ = 0.328, *p* = .567, ${\eta_{p}^{}}^{2}$ = 0.0003, 95% CI = [0–1]). However, the group × sex interactions showed outcomes for the left and right (sub)ACC that were similar to those for the entire ACC (Figure S2).

**Table 2:** (Sub)ACC Cortical Thickness

Group	ACC	dACC	sgACC	pgACC
FE MDD	2.29 (0.51)	2.70 (0.61)	2.06 (0.51)	1.95 (0.45)
Recurrent MDD	2.17 (0.48)	2.58 (0.58)	1.93 (0.44)	1.83 (0.42)
HC	2.31 (0.52)	2.72 (0.61)	2.07 (0.50)	1.96 (0.45)
Group, *p*_uncorr_	.069	.145	.082	.055
Group × Sex, *p*_uncorr_	.013	.095	.006	.003

All cortical thickness values [mean (SD)] are represented in mm.

ACC, anterior cingulate cortex; dACC, dorsal ACC; FE MDD, first-episode major depressive disorder; HC, healthy control participant; pgACC, perigenual ACC; *p*_uncorr_, *p* value uncorrected; sgACC, subgenual ACC.

Compared to the raw CT values, similar results were observed for (sub)ACC CT *z* scores that were derived from the normative model (Figures S3–S5). When groups were matched more equally based on use of medication and research sites, yielding 118 subjects in each group, this did not change the outcome results significantly (Table S2; Figure S6). Notably, there were some differences in (sub)ACC CT lateralization compared to the entire sample; however, the effect sizes (Cohen’s *d*) were quite similar, indicating no major (sub)ACC lateralization differences when groups were matched more equally. In addition, when all 33 adolescents were excluded (20 patients with first-episode MDD [female, *n* = 12; male, *n* = 8], 1 patient with recurrent MDD [male], and 12 HCs [female, *n* = 8; male, *n* = 4]), this did not change the (subregion)ACC CT findings. The outcome also did not change when patients with missing data on the medication intake were included (Table S3; Figure S7). In addition, we included medication in the linear mixed model to examine ACC CT differences between groups on and off medication (excluding HCs). Here also, medication status did not change the outcome results. Therefore, we cannot conclude that medication status affected our ACC CT results (see Supplement, Additional Analysis 1). Finally, when only patients with MDD with moderate to severe depression (HAMD > 17) were included in the analysis, the outcome results did not change (Table S4; Figure S8).

#### Entire ACC

Concerning the entire ACC CT, we found no significant group differences (*F*_2,__19.35_ = 3.074, *p* = .069, ${\eta_{p}^{}}^{2}$ = 0.24, 95% CI = [0–1]). However, we observed a significant group × sex interaction (*F*_2__,__1004.44_ = 4.331, *p* = .013, ${\eta_{p}^{}}^{2}$ = 0.009, 95% CI = [0–1]). Female patients with first-episode MDD had significantly thinner ACC CT compared with their male first-episode depressed counterparts (*p*_FDR_ < .001, Cohen’s *d* = −0.65, 95% CI = [−0.77 to −0.52]). Furthermore, male patients with first-episode MDD showed significantly thicker ACC CT compared with never-depressed male control individuals (*p*_FDR_ = .045, Cohen’s *d* = 0.85, 95% CI = [−0.21 to 1.88]) and male patients with recurrent MDD (*p*_FDR_ = .007, Cohen’s *d* = 1.24, 95% CI = [0.07–2.37]).

#### dACC (BA24)

For the dorsal ACC CT results, we found no significant main effect of group (*F*_2,9.05_ = 2.406, *p* = .145, ${\eta_{p}^{}}^{2}$ = 0.35, 95% CI = [0–1]) and no group × sex interaction (*F*_2,1000.92_ = 2.360, *p* = .095, ${\eta_{p}^{}}^{2}$ = 0.005, 95% CI = [0–1]). Consequently, we did not interpret the post hoc/simple effects tests on lateralization any further.

#### pgACC (BA32)

For the pgACC CT, we found no significant group differences (*F*_2,32.03_ = 3.180, *p* = .055, ${\eta_{p}^{}}^{2}$ = 0.17, 95% CI = [0–1]), but again, we observed a significant group × sex interaction effect (*F*_2,1004.37_ = 5.801, *p*_FDR_ = .009, ${\eta_{p}^{}}^{2}$ = 0.01, 95% CI = [0–1]) revealing that here also female patients with first-episode MDD showed significantly thinner CTs compared with male patients with first-episode depression (*p*_FDR_ < .001, Cohen’s *d* = −0.65, 95% CI = [−0.78 to −0.52]). Male patients with recurrent MDD had thinner pgACC CT compared with male never-depressed HCs (*p*_FDR_ = .036, Cohen’s *d* = −0.81, 95% CI = [−1.79 to 0.18]) and male patients with first-episode MDD (*p*_FDR_ = .002, Cohen’s *d* = −1.4, 95% CI = [−2.56 to −0.20]). In addition, the latter group showed significantly thicker pgACC CT compared with male HCs (*p*_FDR_ = .036, Cohen’s *d* = 0.81, 95% CI = [−0.25 to 1.85]).

#### sgACC (BA25)

Concerning the sgACC CT, we again found no significant group differences (*F*_2,23.93_ = 2.780, *p* = .082, ${\eta_{p}^{}}^{2}$ = 0.19, 95% CI = [0–1]). However, we observed a significant group × sex interaction (*F*_2,999.55_ = 5.111, *p*_FDR_ = .009, ${\eta_{p}^{}}^{2}$ = 0.01, 95% CI = [0–1]) showing that female patients with first-episode MDD showed significantly thinner sgACC CT compared with their male first-episode depressed counterparts (*p*_FDR_ < .001, Cohen’s *d* = −0.73, 95% CI = [−0.85 to −0.60]). Similarly, male patients with recurrent MDD had thinner sgACC CT compared with male patients with first-episode MDD (*p*_FDR_ = .010, Cohen’s *d* = −1.71, 95% CI = [−2.29 to −0.01]), and again male patients with first-episode MDD showed a thicker CT compared to the male HC group (*p*_FDR_ = .010, Cohen’s *d* = 1.05, 95% CI = [−0.03 to 2.10]).

## Discussion

Several observations were made based on the findings of this structural brain imaging project: 1) in general, women displayed significantly thinner ACC CT compared with men; 2) sex influenced first-episode and recurrent MDD differently; 3) CT reductions were mainly observed in the “emotional” ACC subdivisions (pgACC, sgACC) but not in the “cognitive” dACC; and 4) no (sub) ACC CT lateralization differences were detected. Moreover, similar findings were observed with a more individualized approach, which is an additional validation of the findings (Figures S3–S5). It is also important to note that depression severity, whether or not medication was taken, matching for equal groups, and including adults only did not significantly change the ACC CT outcome results (see Tables S2–S4 and Figures S6–S8, and Supplement, Additional Analysis 1).

Overall, our findings that women showed significantly thinner ACC CT compared with men seem to be the opposite of the earlier findings of Luders *et al.* (14), who showed significantly greater whole-brain CT in women compared with men. Besides that, only a relatively small sample of healthy individuals was included, and methodological differences between the studies—with the most relevant one being the exclusion of the ACC CT—makes it difficult to compare. Although this difference in ACC CT may be attributed to greater variability in regional brain structure in males than in females or that in general the male brain is larger than the female brain (17), it is believed that genetic or gene-environment interaction mechanisms related to sex early in life may lead to specific differences in brain structure (47).

Concerning our major research questions, female patients with first-episode MDD had significantly thinner ACC CT compared with their first-episode depressed male counterparts. Because the sgACC and the pgACC showed a similar CT pattern but the dorsal parts of the ACC did not, a first logical deduction could be that stress sensitivity, fluctuations in sex hormones, and influences on inflammatory parameters may account for the effects. Indeed, it has been shown that hyperactivity of the hypothalamic-pituitary-adrenal system can lead not only to increased proinflammatory activity with impaired synaptogenesis and neuronal death (48), but it can also increase anxiety in women (9,49, 50, 51), a symptom that is frequently observed in females with depression (3). Moreover, especially in women, it has been suggested that reduced ACC CT and low vagal-mediated resting heart rate variability, an indicator of poorer stress regulation, could serve as high-risk endophenotypes for the development of MDD (27). Although this research dataset did not include physiological parameters, our findings suggest that the structural impact of depressive illness in females has already become significant by the time of their first episode and that the lack of ACC CT normalization may keep this group vulnerable to relapse (22).

In contrast, male patients with first-episode MDD showed significantly thicker ACC CT compared with healthy male controls as well as male patients with recurrent MDD. Again, this substantiates former assumptions that cortical morphometry characteristics in MDD are highly sex-specific and may occur early (at illness onset) (16). Although such discrepancies may also be related to differences in the expression of depressive symptoms in men versus women (2) or to sex differences in hypothalamic-pituitary-adrenal system (re)activity (52), the observed higher CT in male patients with first-episode MDD—although speculative—may represent a compensatory, neuroplastic response in an effort to attenuate depressive symptomatology (53). Li *et al.* suggested that CT increases may result from the dual activation of the immune-inflammatory response system and the compensatory immune-regulatory reflex system, resulting in preapoptotic osmotic changes, cellular hypertrophy, and cytokine-activated astrocyte proliferation (15). Our current findings that ACC CT increases in male patients with first-episode MDD are consistent with the assumptions of Li *et al.* that such presumed compensatory mechanisms may be particularly relevant during the early stages of MDD because CT and activity seem to be increased in some regions early in the illness course but decrease as the illness progresses. Despite the fact that we did not include neuroinflammation parameters, our current data add to these assumptions that differences in ACC CT and its subregions are driven by neurobiological differences between men and women.

Finally, consistent with ENIGMA (Enhancing Neuro Imaging Genetics through Meta Analysis) MDD Working Group conclusions stating that cortical brain areas may be differentially affected (54), in the current study, only the ACC “emotional” and not the “cognitive” subdivisions were affected. Of course, it may still be possible that the dACC may become affected as well in certain subtypes of depression (55,56), in patients with a history of childhood maltreatment (57), when exposed to prolonged and excessive exposure to stress (glucocorticoids) (58), or when confronted with treatment resistance (59). Indeed, such a long-term neurotoxic environment leads to impaired neurogenesis, neuronal loss, and loss of brain tissue (48). Although lateral differences in ACC neuronal functions were documented (60), our findings are also consistent with the results of the ENIGMA-Laterality Working Group and others where no significant changes in CT asymmetry were demonstrated in MDD (61,62). However, structure does not equal function, and functional sgACC lateralization differences in patients with clinical depression have been documented (34).

Given the known differences in symptom representation between sexes and the fact that functional outcomes may differ between men and women (4), sex-specific (psycho)therapeutic interventions that target dysfunctional emotion regulation processes may be warranted. Indeed, determinants of antidepressant treatment response and remission may be mediated in a sex- and drug-class-specific manner (63). Our ACC CT findings suggest that such interventions are best initiated early during the first depressive episode. Noninvasive brain stimulation methods such as repetitive transcranial magnetic stimulation affecting sgACC processes may be of interest when patients do not reach full remission (with elevated risk to relapse) or are treatment-resistant (64, 65, 66, 67). Although it is still unclear, women and men may respond differently to this type of treatment (68,69).

Although this was not considered a major constraint, all the recruitment sites were in Asia (China). Due to the unavailability of some important information in the REST-meta-MDD database, we could not consider the influence of the number of depressive episodes, the duration of treatment, the age at which the first depressive episode started (for the recurrent group), or the occurrence of early adverse events. The number of patients with first-episode MDD was considerably higher than the number with recurrent MDD. In essence, this is a cross-sectional dataset and not a longitudinal study in which the same patients with MDD were followed up. Notably, we included patients with a confirmed diagnosis of MDD, but because information on the type of depression was not available, we could not distinguish between subtypes of depression (e.g., MDD with melancholic or anxious features) nor whether recurrent depressed patients were treatment-resistant or not. Although we only focused on ACC CT, our findings do not imply that other key brain areas and/or networks would not be affected during the course of depressive illness (15,54). One could argue that the women in our study cohort had a higher mean age than men, thereby explaining the thinner ACC CT in the groups. However, the mean ages of the men and women in our study were very similar (Table 1), and we adjusted for age in our analysis, making it unlikely that small but significant age differences between the sexes would be responsible for this main effect. Finally, the fact that we lacked access to additional sex-specific information or other physiological variables related to sex should be considered a limitation of this study.

### Conclusions

In summary, sex determined ACC CT over the course of MDD differently. ACC cortical changes were primarily observed in those ACC subdivisions that are associated with dysregulation of emotions. These observations underline the importance of effective clinical interventions for stress regulation at an early stage in the course of depressive illness and investing more in relapse prevention.

## Supplementary Material

Supplementary Data

Key Resources Table
