# Supplementary material for: Sex Determines Anterior Cingulate Cortex Cortical Thickness in the Course of Depression
Source: Biol Psychiatry Glob Open Sci. 2023 Aug 13;4(1):346–53. doi: 10.1016/j.bpsgos.2023.08.003 (PMC11639738; doi:10.1016/j.bpsgos.2023.08.003)
Supplement: Supplementary Data [file mmc1.pdf]

SUPPLEMENTARY INFORMATION

Sex Determines Anterior Cingulate Cortex Cortical Thickness in the Course of Depression

Wu and Baeken

	ACC.L	ACC.R	dACC.L	dACC.R	sgACC.L	sgACC.R	pgACC.L	pgACC.R
FE MDD	2.35 (0.52)	2.24 (0.51)	2.84 (0.64)	2.59 (0.61)	2.02 (0.53)	2.10 (0.52)	1.97 (0.45)	1.92 (0.47)
Recurrent MDD	2.20 (0.49)	2.14 (0.50)	2.70 (0.62)	2.48 (0.61)	1.90 (0.47)	1.96 (0.45)	1.81 (0.41)	1.84 (0.45)
HC	2.37 (0.55)	2.25 (0.51)	2.86 (0.70)	2.60 (0.63)	2.04 (0.53)	2.09 (0.50)	1.98 (0.47)	1.94 (0.46)
P_{uncorr}(Group)	0.050	0.114	0.149	0.184	0.097	0.090	0.016	0.456
P_{uncorr}(Group ×Sex)	0.010	0.027	0.032	0.300	0.023	0.004	0.007	0.003

Supplemental Table 1. Cortical thickness of the bilateral ACC and its subregions across diverse groups.

Group	Sex	Count	Age (Mean/SD)	Education (Mean/SD)	Medication (Yes/No)	HAMD (Mean/SD)
FE MDD	/	118	37.01 (12.21)	11.12 (3.70)	78/40	22.05 (4.41)
	Female	78	38.59 (12.12)	10.53 (3.92)	55/23	22.59 (4.02)
	Male	40	33.92 (11.94)	12.28 (2.93)	23/17	21.00 (4.96)
Recurrent MDD	/	118	39.98 (13.26)	10.43 (3.87)	81/37	22.86 (5.30)
	Female	77	42.04 (12.69)	10.36 (3.97)	50/27	23.22 (5.19)
	Male	41	36.12 (13.59)	10.56 (3.73)	31/10	22.20 (5.49)
HC	/	118	43.46 (13.54)	12.03 (4.20)	/	/
	Female	70	46.53 (12.83)	11.46 (4.11)	/	/
	Male	48	38.98 (13.43)	12.88 (4.23)	/	/
p / η_p^2 (Group)		/	0.254 / 0.25	0.003[c] / 0.35	0.781[a]	0.342 / 0.06
p / η_p^2 (Sex)		/	0.003[b] / 0.03	0.016 / 0.02	0.983[a]	0.145 / 0.009
p / η_p^2 (Group×Sex)		0.499[a]	0.79 / 0.001	0.720 / 0.009	/	0.803 / 0.0003

Supplemental Table 2. Demographics matched with MatchIt R package for medication usage.

FE MDD: first episode major depressive disorder; HC: healthy controls; [a]: Chi-square test of independence; [b]: Females have a higher mean age than males; [c]: Post hoc Tukey's HSD tests indicated that educational attainment was higher in the HC group relative to the recurrent MDD group (t=3.42, $p_{adjusted}$ = 0.039).

Group	Sex	Count	Age (Mean/SD)	Education (Mean/SD)	Medication (Yes/No/Unknow)	HAMD (Mean/SD)
FE MDD	/	415	34.92 (12.38)	11.29 (3.71)	131/250/34	22.56 (4.71)
	Female	279	35.63 (12.32)	11.19 (3.72)	94/165/20	22.91 (4.60)
	Male	136	33.48 (12.42)	11.48 (3.68)	37/85/14	21.84 (4.87)
Recurrent MDD	/	162	38.56 (13.39)	10.84 (3.66)	81/37/44	22.33 (5.20)
	Female	98	40.55 (12.96)	10.63 (3.81)	50/27/21	22.71 (5.27)
	Male	64	35.50 (13.57)	11.16 (3.41)	31/10/23	21.75 (5.08)
HC	/	606	36.61 (13.92)	12.87 (3.99)	/	/
	Female	371	37.75 (14.24)	12.48 (4.12)	/	/
	Male	235	34.80 (13.22)	13.48 (3.70)	/	/
p / η_p^2 (Group)		/	0.313 / 0.08	0.002[c] / 0.76	<0.001[a]	0.985 / 0.000008
p / η_p^2 (Sex)		/	0.004[b] / 0.007	0.056 / 0.003	0.038[a]	0.074 / 0.006
p / η_p^2 (Group×Sex)		0.109[a]	0.824 / 0.0003	0.185 / 0.003	/	0.657 / 0.0004

Supplemental Table 3. Demographics when all patients are included, also with the status medication unknown.

FE MDD: first episode major depressive disorder; HC: healthy controls; [a]: Chi-square test of independence; [b]: Females have a higher mean age than males; [c]: Post hoc Tukey's HSD tests indicated that educational attainment was higher in the HC group compared to both the recurrent MDD group (t=3.45, $p_{\text{adjusted}} = 0.03$) and FE MDD group (t=3.43, $p_{\text{adjusted}} = 0.042$).

Group	Sex	Count	Age (Mean/SD)	Education (Mean/SD)	Medication (Yes/No)	HAMD (Mean/SD)
FE MDD	/	341	35.26 (12.28)	11.48 (3.74)	117/224	23.46 (4.39)
	Female	239	35.43 (12.18)	11.33 (3.80)	86/153	23.55 (4.36)
	Male	102	34.87 (12.55)	11.84 (3.60)	31/71	23.24 (4.47)
Recurrent MDD	/	107	40.00 (13.14)	10.50 (3.84)	72/35	23.61 (4.98)
	Female	69	41.81 (12.42)	10.45 (3.91)	43/26	24.07 (4.78)
	Male	38	36.71 (13.93)	10.58 (3.76)	29/9	22.76 (5.29)
HC	/	524	36.77 (14.18)	12.89 (4.18)	/	/
	Female	329	37.79 (14.45)	12.51 (4.26)	/	/
	Male	195	35.05 (13.57)	13.54 (3.96)	/	/
p / η_p^2 (Group)		/	0.644 / 0.05	<0.001[b] / 0.70	<0.001[a]	0.835 / 0.0003
p / η_p^2 (Sex)		/	0.108 / 0.003	0.161 / 0.002	0.928[a]	0.205 / 0.004
p / η_p^2 (Group×Sex)		0.085[a]	0.607 / 0.001	0.271 / 0.003	/	0.407 / 0.002

Supplemental Table 4. Demographics when MDD patients are included with HAMD>17.
FE MDD: first episode major depressive disorder; HC: healthy controls; [a]: Chi-square test of independence; [b]: Post hoc Tukey's HSD tests indicated that educational attainment was higher in the HC group relative to the recurrent MDD group (t=3.55, $p_{\text{adjusted}} = 0.041$).

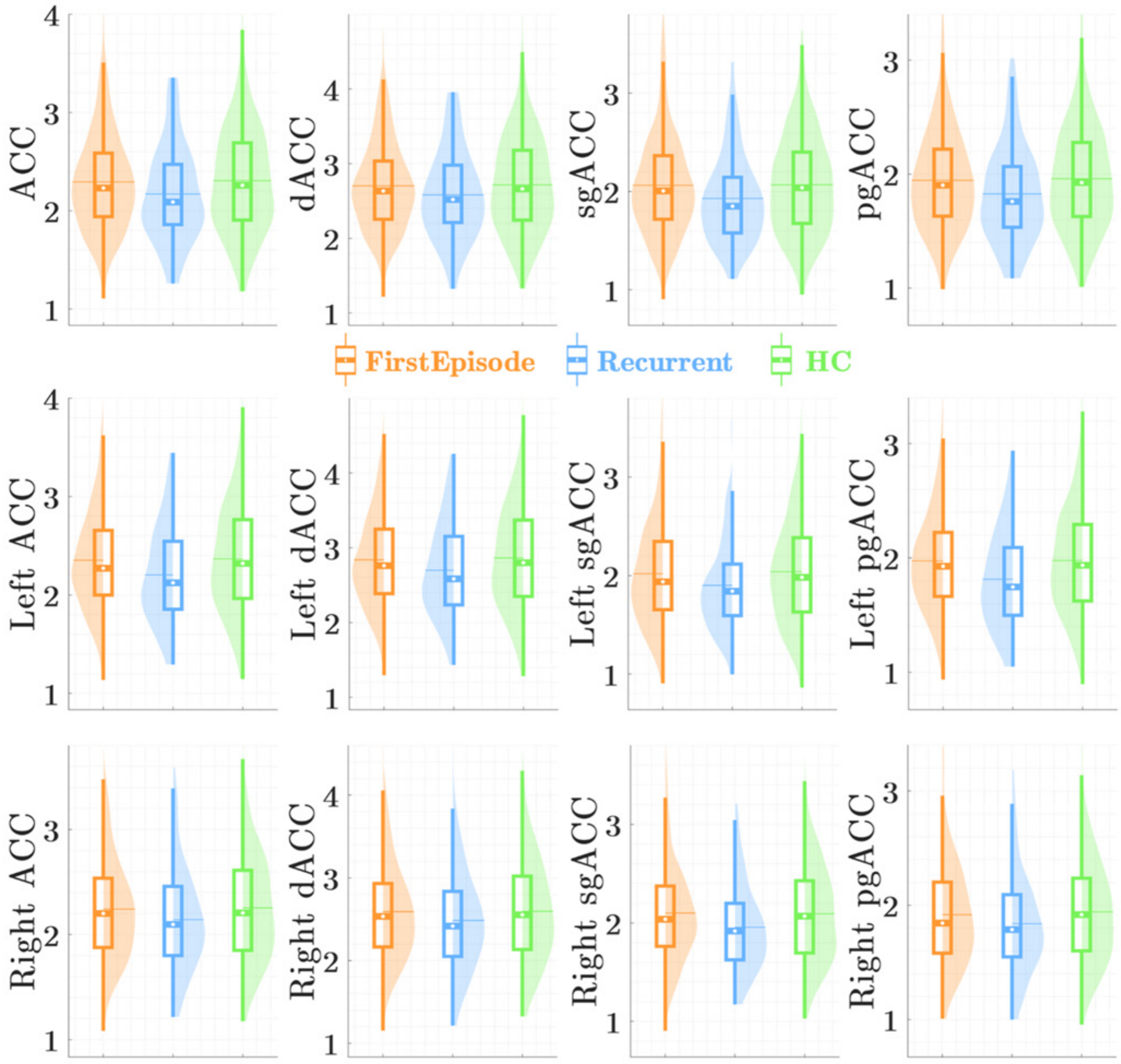

Supplemental Figure 1. Distribution of (sub)ACC cortical thickness across Groups.

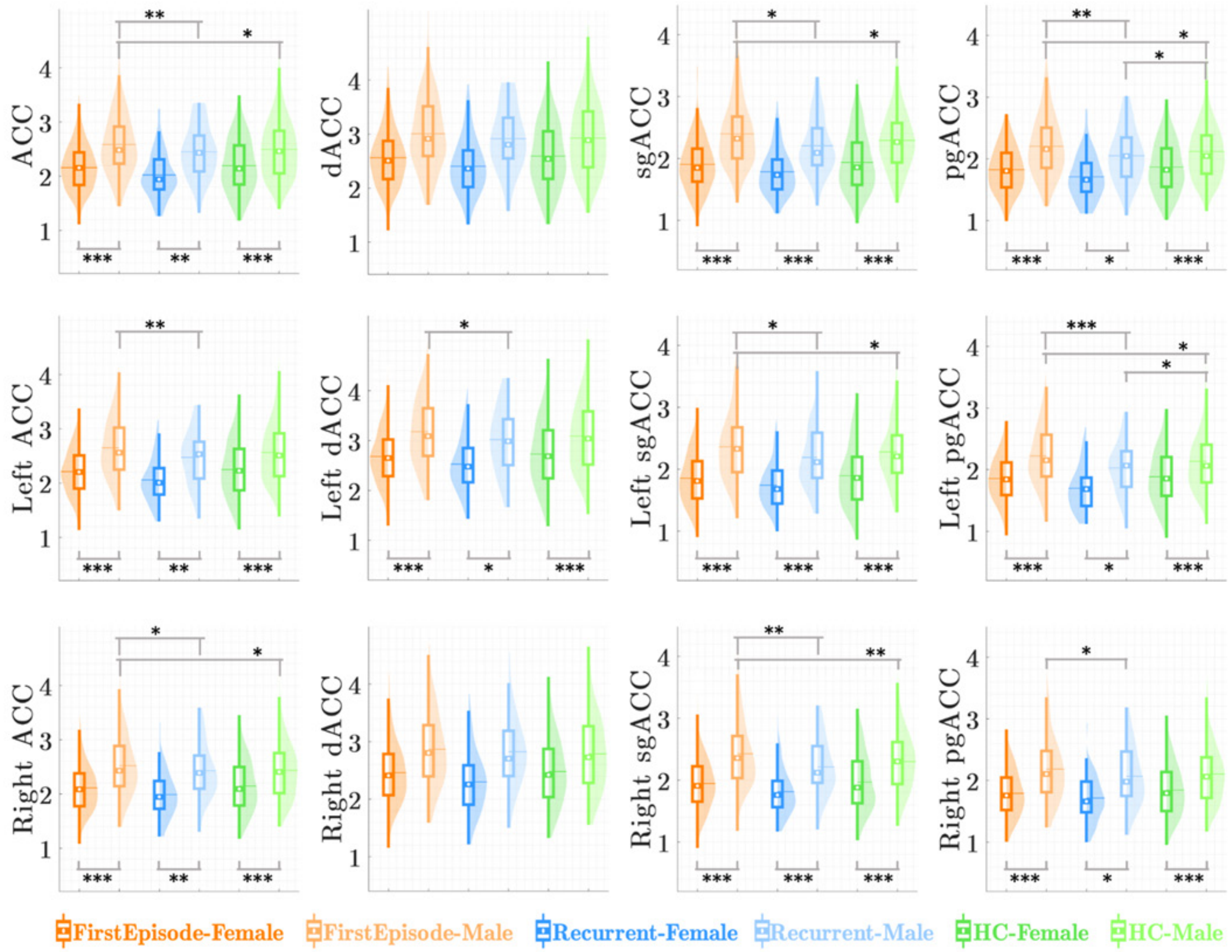

Supplemental Figure 2. Distribution of ACC cortical thickness across Groups and Sex. FDR correction was applied for the simple effects tests of Group×Sex interaction. (* $p < 0.05$, ** $p < 0.01$, *** $p < 0.001$).

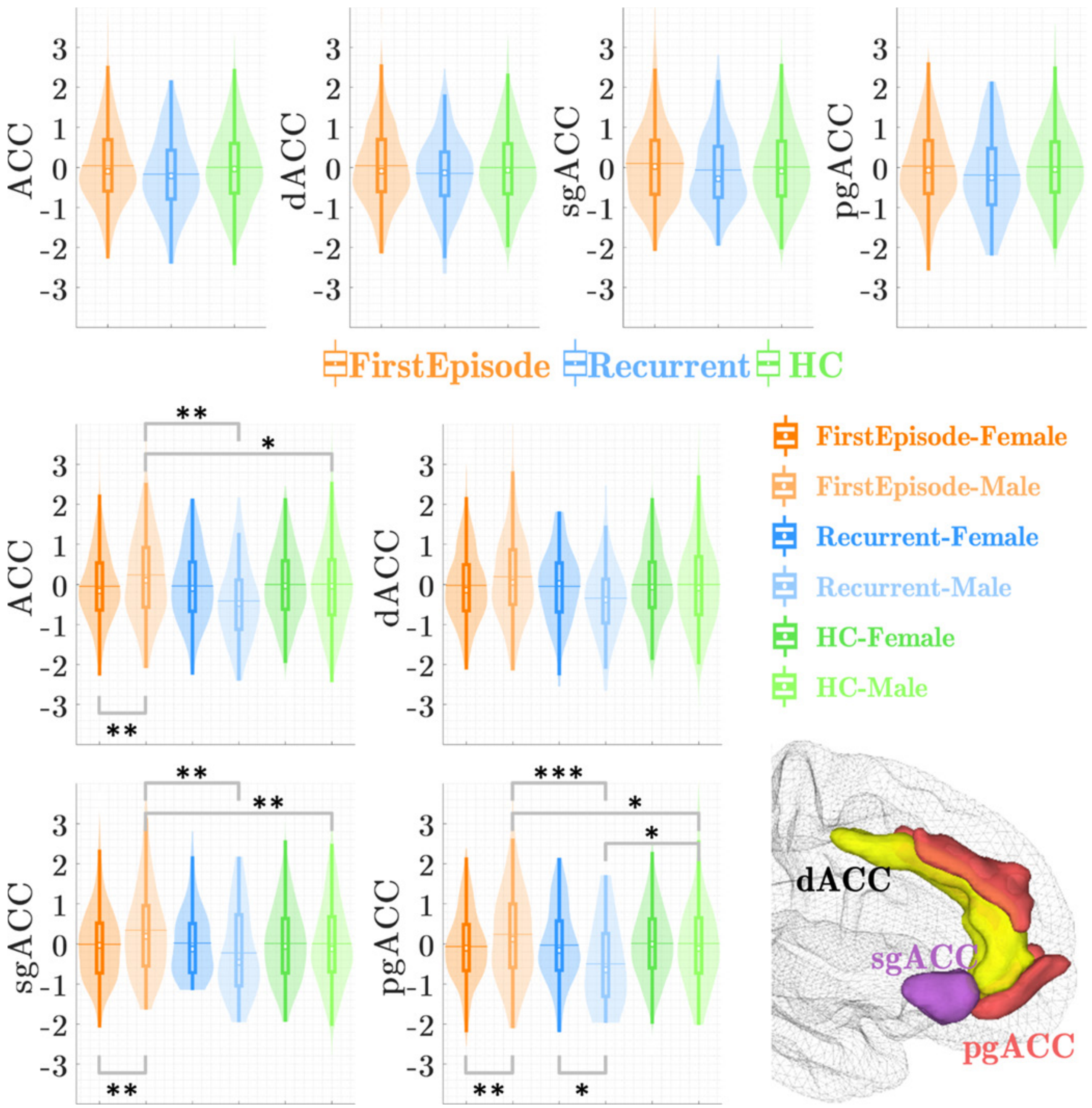

Supplemental Figure 3. Distribution of ACC cortical thickness *z*-scores across Groups and Sex. FDR correction was applied for the simple effects tests of Group×Sex interaction. (* $p < 0.05$, ** $p < 0.01$, *** $p < 0.001$).

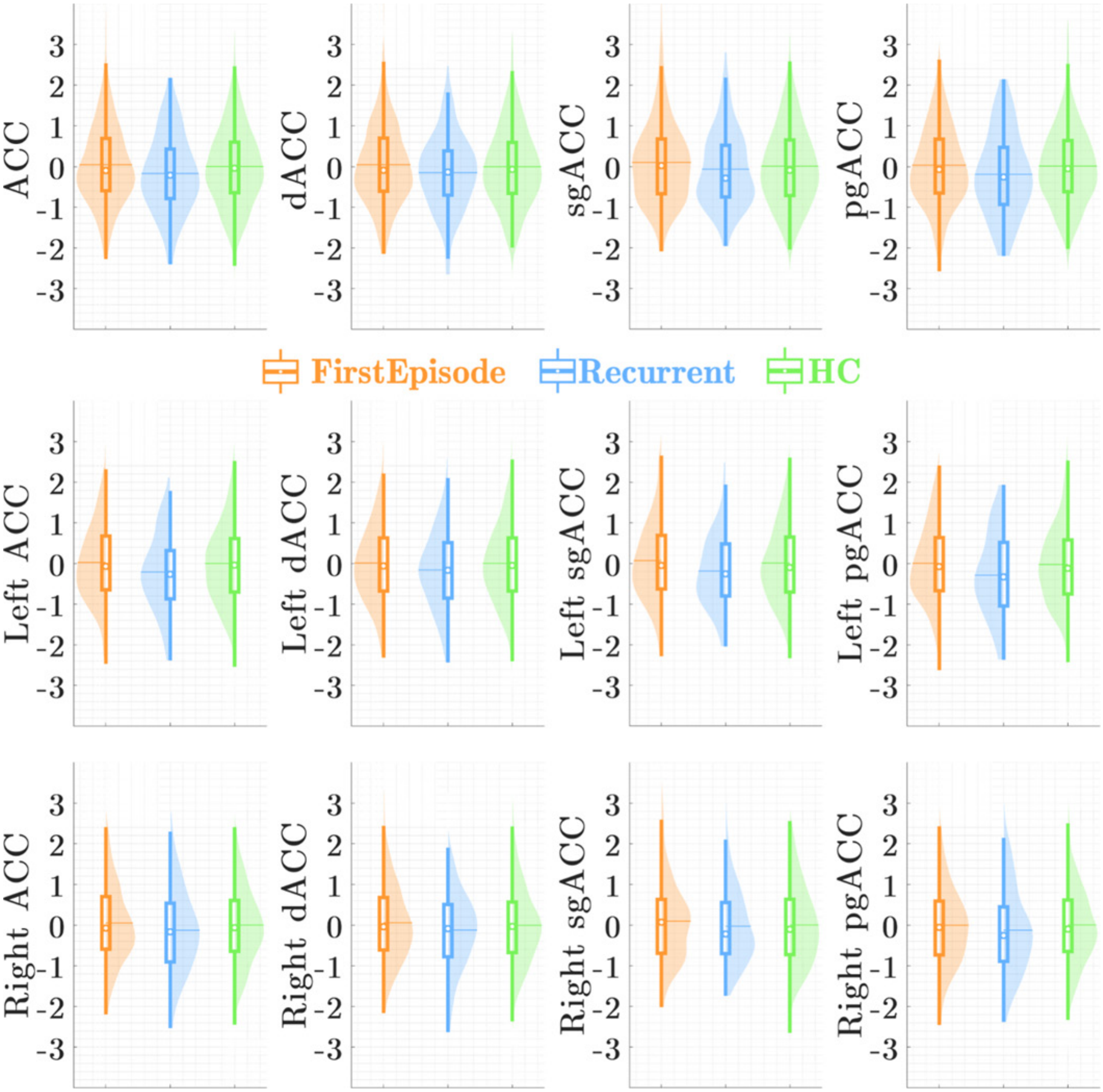

Supplemental Figure 4. Distribution of ACC cortical thickness z-scores across Groups.

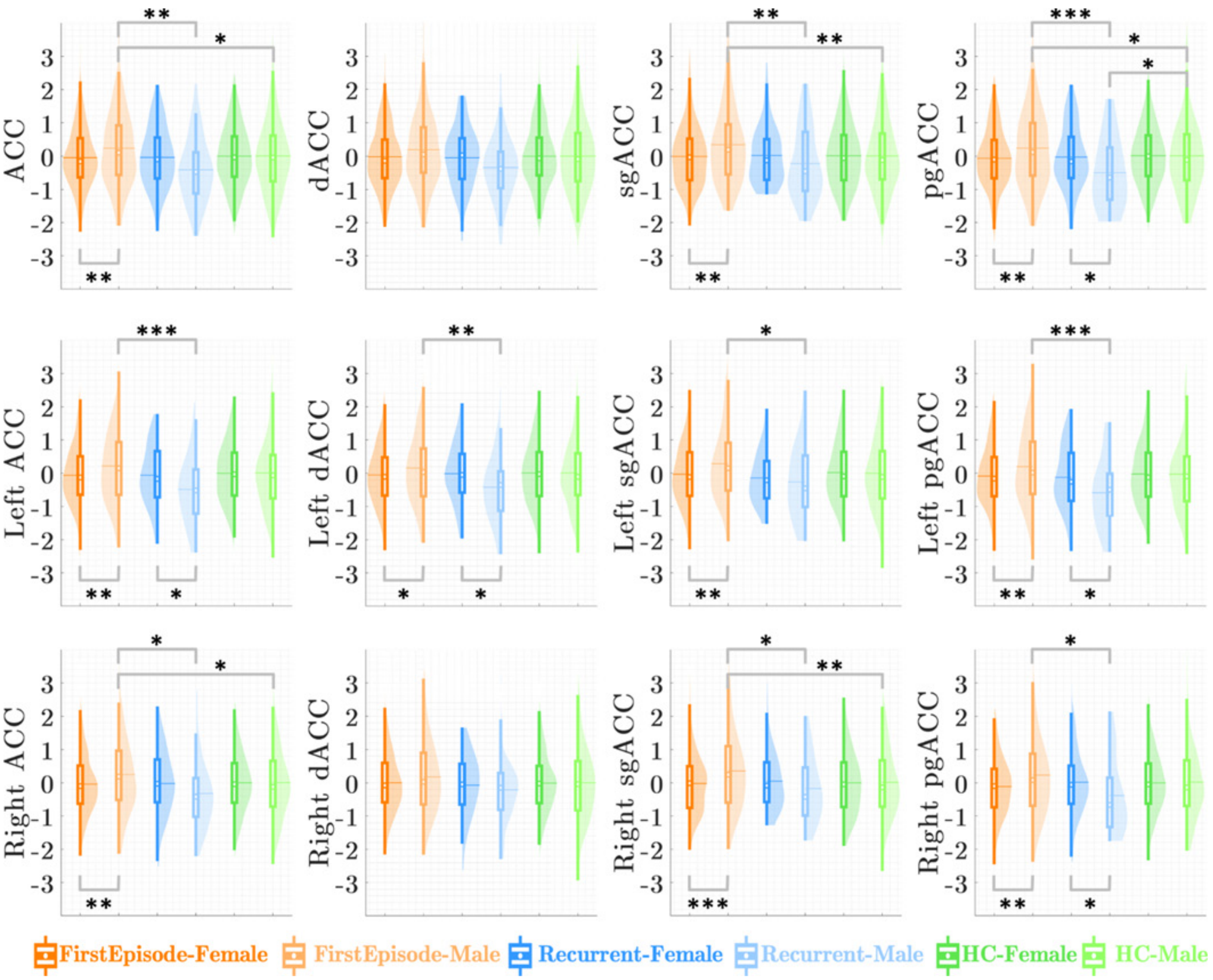

Supplemental Figure 5. Distribution of ACC cortical thickness z-scores across Groups and Sex. FDR correction was applied for the simple effects tests of Group×Sex interaction. (* $p < 0.05$, ** $p < 0.01$, *** $p < 0.001$).

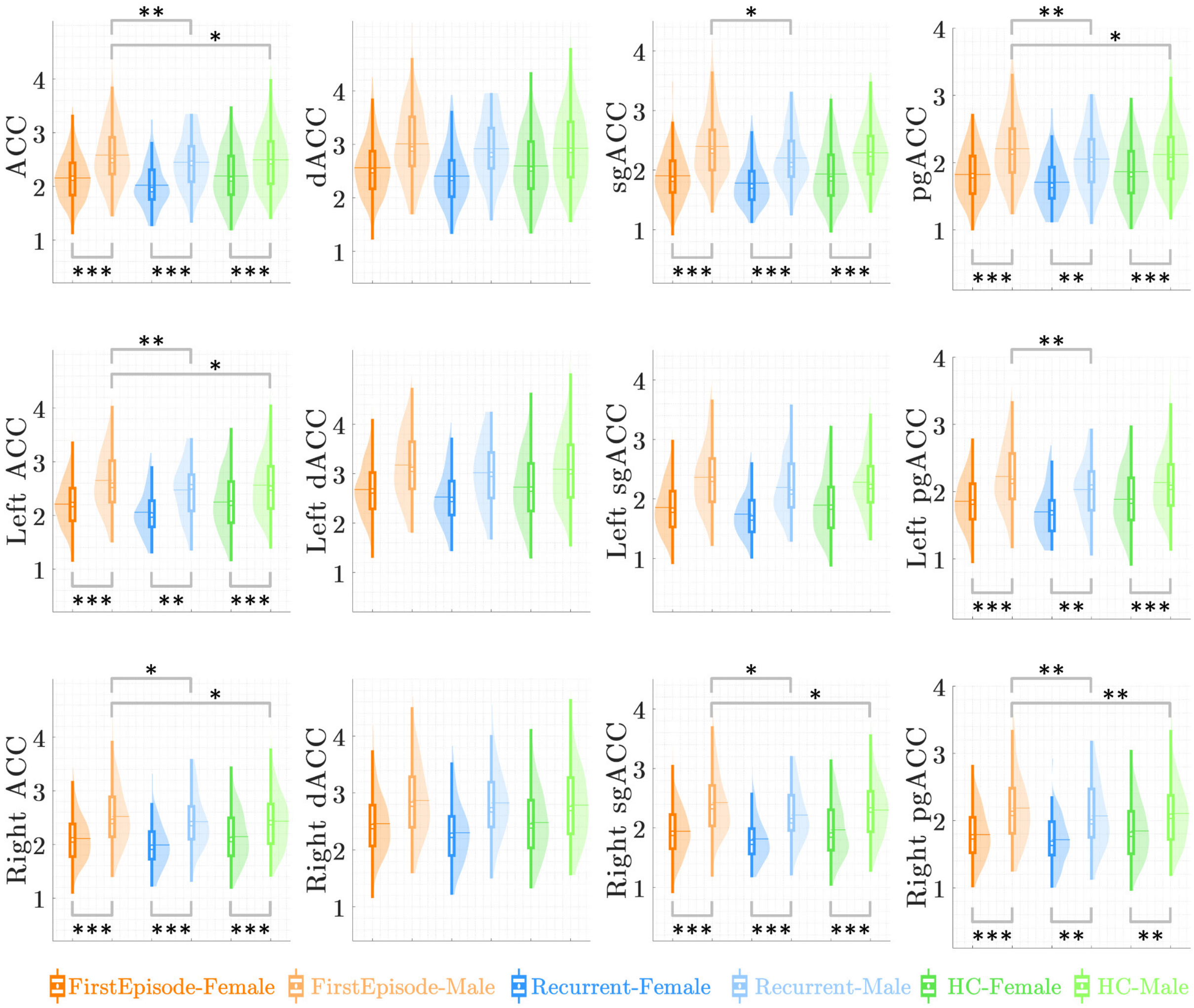

Supplemental Figure 6. Distribution of ACC cortical thickness across Groups and Sex. FDR correction was applied for the simple effects tests of Group×Sex interaction. (* $p < 0.05$, ** $p < 0.01$, *** $p < 0.001$). Subjects were matched using the MatchIt R package (matching FE vs. Recurrent MDD vs HC based on the use of medication and research sites). This yielded 118 subjects in each group.

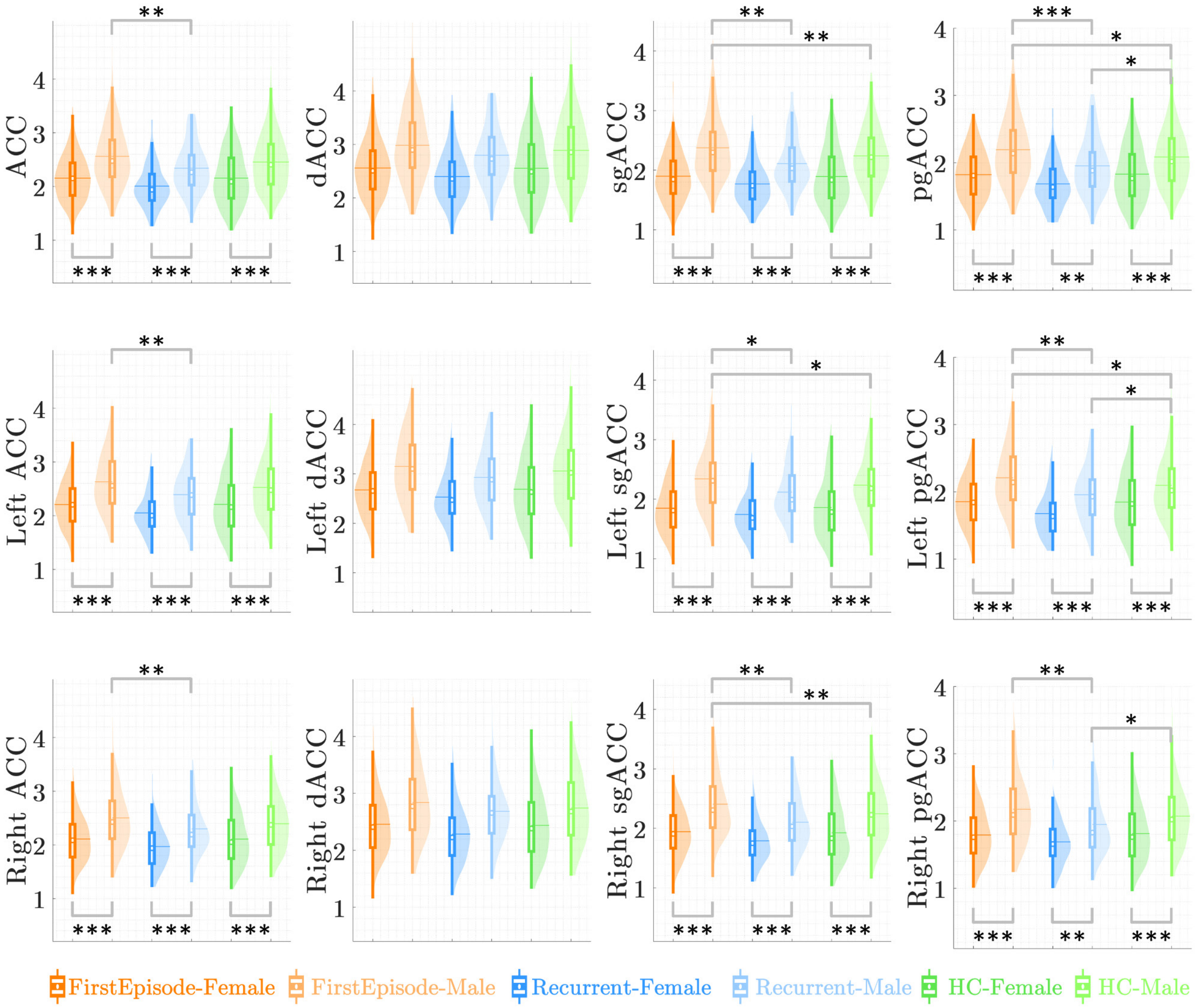

Supplemental Figure 7. Distribution of ACC cortical thickness across Groups and Sex. FDR correction was applied for the simple effects tests of Group×Sex interaction. (* $p < 0.05$, ** $p < 0.01$, *** $p < 0.001$). When including patients with missing data on the medication intake, 415 first episode MDD patients (male=136; female=279) and 162 recurrent MDD patients (male=64; female=98) were included.

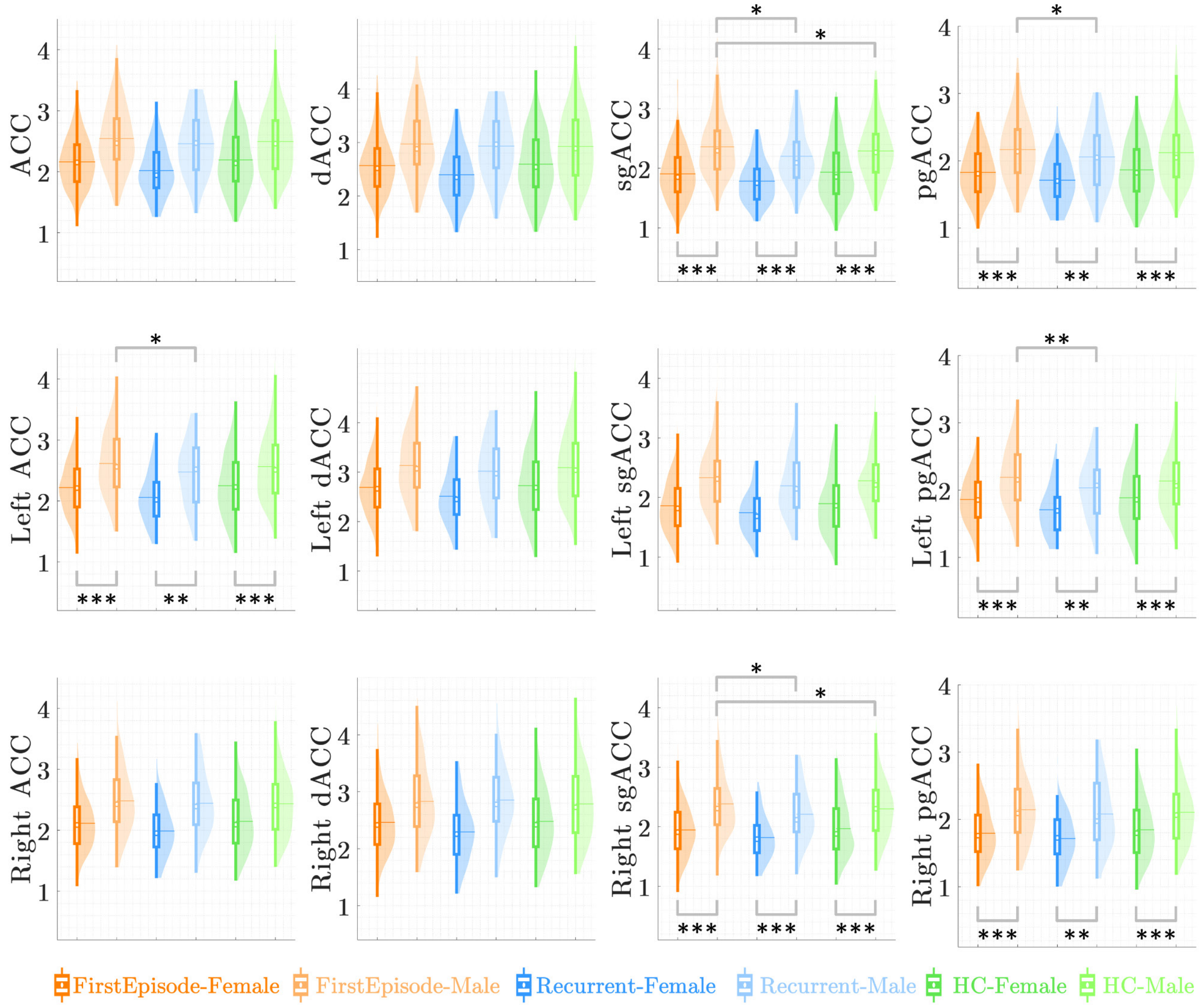

Supplemental Figure 8. Distribution of ACC cortical thickness across Groups and Sex. FDR correction was applied for the simple effects tests of Group×Sex interaction. (* $p < 0.05$, ** $p < 0.01$, *** $p < 0.001$). When including only MDD patients with a HAMD score>17, 341 first episode MDD patients (male=102; female=239) and 107 recurrent MDD patients (male=38; female=69) were included.

Additional analysis 1. We included medication in the linear mixed model (shown below) to examine whether there were ACC CT differences between groups on and off medication (excluding HC). Medication status did not change the outcome results. Therefore, we cannot conclude that medication status affected our ACC CT results.

The following analyses were carried out.

ACC CT ~ 1 + Medication* Group*Sex + Age + Edu+ (1 + Group | Site).

Where '*' is the Wilkinson-Rogers notation, Medication*Group*Sex indicates Medication+Group+Sex+Group:Medication+Sex:Medication+Group:Sex+Group:Medication:Se x

For the raw sample (381 first episode MDD, 118 recurrent MDD, and 524 healthy controls (HC)), no significant ACC CT differences were found related to medication intake or not *(p*=0.542, η_p^2=0.0009), group:medication interactions(*p*=0.553, η_p^2=0.0008), medication:sex interactions (*p*=0.841, η_p^2=0.00009), and group:medication:sex interactions (*p*=0.602, η_p^2=0.0006). Crucially, the group:sex interaction was still significant (*p*=0.023, η_p^2=0.01).

For the matched sample (118 in each group), no significant ACC CT differences was found related to medication (*p*=0.088, η_p^2=0.01), group:medication interactions(*p*=0.514, η_p^2=0.002), medication:sex interactions(*p*=0.748, η_p^2=0.0005), and group:medication:sex interactions (*p*=0.798, η_p^2=0.0003). Crucially again, the group:sex interaction remained significant (*p*=0.005, η_p^2=0.03).
